# Supplementary figures and images for: DRUL for school: Opening Pre-K with safe, simple, sensitive saliva testing for SARS-CoV-2
Source: PLoS One. 2021 Jun 25;16(6):e0252949. doi: 10.1371/journal.pone.0252949 (PMC8232451; doi:10.1371/journal.pone.0252949)

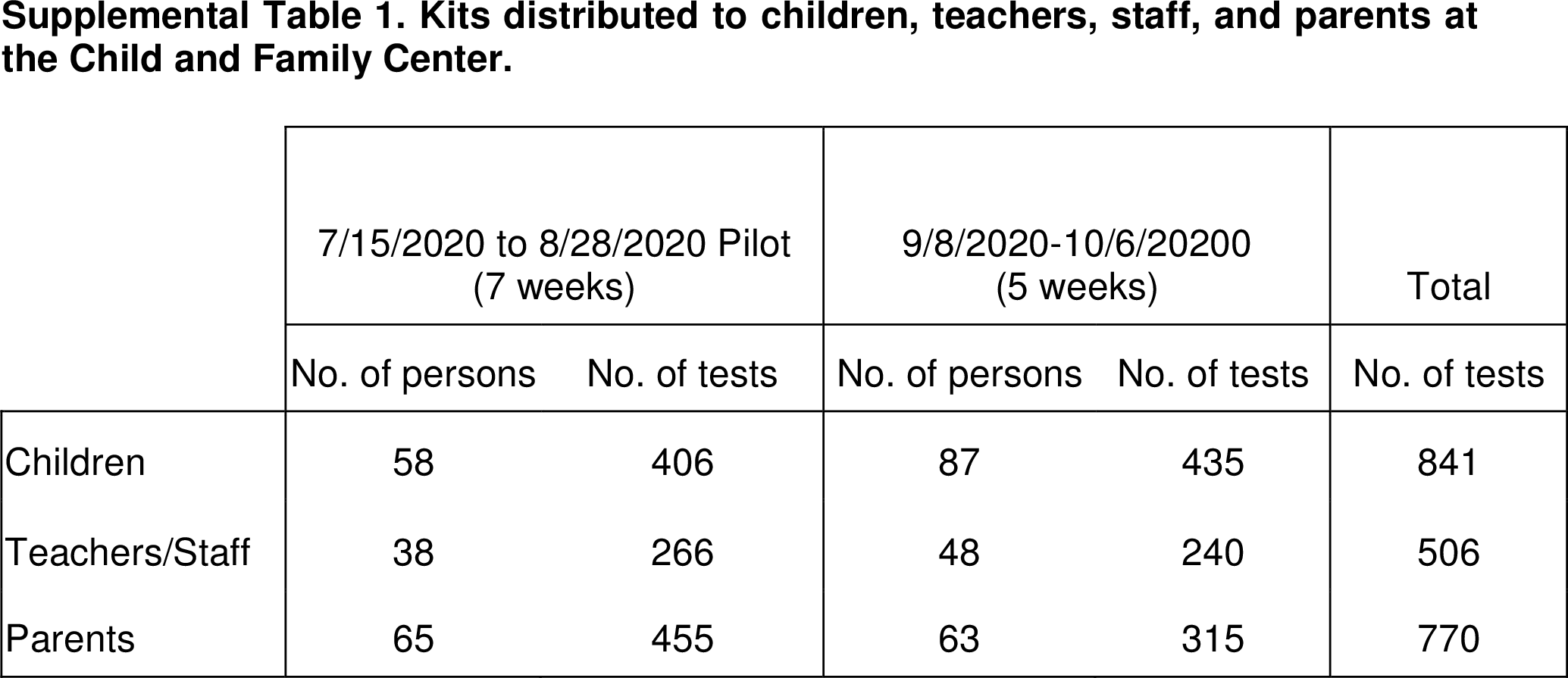

Supplement: S1 Table — (TIF) [file pone.0252949.s001.tif]

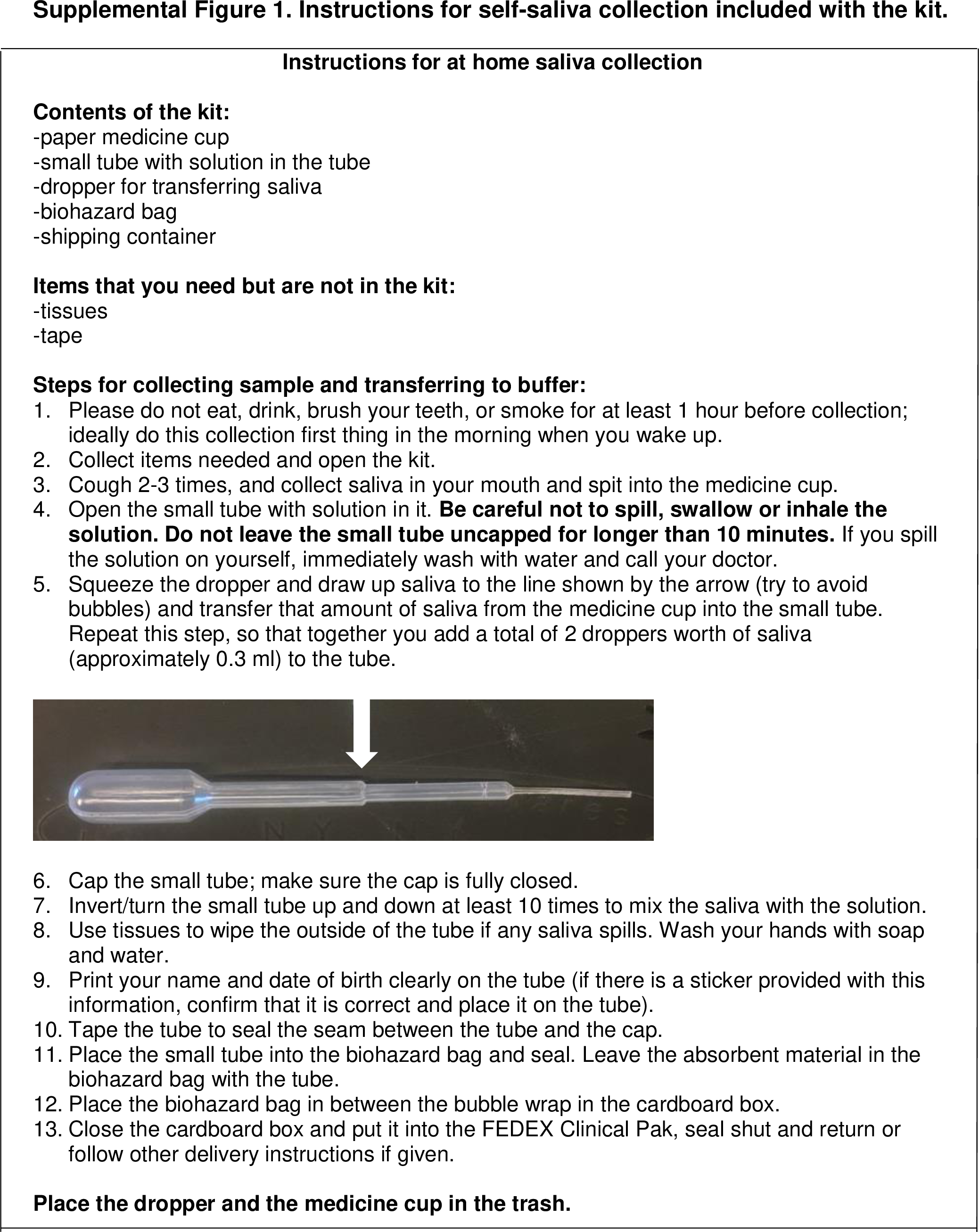

Supplement: S1 Fig — (TIF) [file pone.0252949.s002.tif]
